# Defining upper gastrointestinal bleeding from linked primary and secondary care data and the effect on occurrence and 28 day mortality

**DOI:** 10.1186/1472-6963-12-392

**Published:** 2012-11-13

**Authors:** Colin John Crooks, Timothy Richard Card, Joe West

**Affiliations:** 1 Division of Epidemiology and Public Health, The University of Nottingham, Clinical Sciences Building 2, City Hospital, Nottingham, NG5 1PB, UK; 2 Nottingham Digestive Diseases Centre, National Institute for Health Research Biomedical Research Unit, Queen’s Medical Centre, Nottingham University Hospitals National Health Service Trust, Nottingham, NG7 2UH, UK

**Keywords:** Selection bias, Mortality, Data linkage, Upper gastrointestinal bleeding, Case definitions

## Abstract

**Background:**

Primary care records from the UK have frequently been used to identify episodes of upper gastrointestinal bleeding in studies of drug toxicity because of their comprehensive population coverage and longitudinal recording of prescriptions and diagnoses. Recent linkage within England of primary and secondary care data has augmented this data but the timing and coding of concurrent events, and how the definition of events in linked data effects occurrence and 28 day mortality is not known.

**Methods:**

We used the recently linked English Hospital Episodes Statistics and General Practice Research Database, 1997–2010, to define events by; a specific upper gastrointestinal bleed code in either dataset, a specific bleed code in both datasets, or a less specific but plausible code from the linked dataset.

**Results:**

This approach resulted in 81% of secondary care defined bleeds having a corresponding plausible code within 2 months in primary care. However only 62% of primary care defined bleeds had a corresponding plausible HES admission within 2 months. The more restrictive and specific case definitions excluded severe events and almost halved the 28 day case fatality when compared to broader and more sensitive definitions.

**Conclusions:**

Restrictive definitions of gastrointestinal bleeding in linked datasets fail to capture the full heterogeneity in coding possible following complex clinical events. Conversely too broad a definition in primary care introduces events not severe enough to warrant hospital admission. Ignoring these issues may unwittingly introduce selection bias into a study’s results.

## Background

Electronic health records of routinely recorded data are increasingly used in health research. They are relatively cheap, convenient, and provide power for studies that would be unfeasible in bespoke patient cohorts. Previously our group has used routine secondary care data (Hospital Episodes Statistics - HES) to define incidence and deprivation [1], and mortality trends [2] for upper gastrointestinal bleeding and we reassuringly found that the numbers of cases and procedures identified using HES were comparable to a national hospital audit [3]. However for future studies investigating aetiological factors we require comprehensive prescription and co-morbidity data for each patient prior to their hospital admission. As this was either unavailable or incomplete in secondary care data we planned to use primary care data (General Practice Research Database - GPRD) in which the coding for upper gastrointestinal bleeding was shown to be valid in 99% of cases by chart review [4]. To retain the advantages in using secondary care data of procedural coding, multiple hospital diagnoses, and accurate admission dates, we took the opportunity to use linked GPRD and HES data.

However our initial attempts to define a linked cohort of upper gastrointestinal bleeding demonstrated discrepancies in the cases detected between primary care and secondary care. We have therefore investigated the reasons for this by studying alternative methods of defining cases (separately in each dataset or various combinations from both datasets) and to what extent the choice between these methods will affect our results.

## Methods

### Databases

#### Hospital episodes statistics

HES contains information on all admissions to an NHS hospital in England, with over 12 million new records added each year [5]. Each admission will have up to 20 diagnoses coded using the International Classification of Diseases 10th revision (ICD 10); and up to 24 procedures coded using the United Kingdom Tabular List of the Classification of Surgical Operations and Procedures (version OPCS4).

#### General practice research database

The GPRD contains longitudinal primary care data coded using the Read code system that are validated and individualised for over 46 million person years since 1987 [6]. The data are subject to quality checks and when the data are of high enough quality to be used in research they are referred to as “up to standard.” The GPRD has been extensively validated for a wide range of chronic diagnoses and consistently found to be accurate [7-9]. This study was part of an ethical approval from the Independent Scientific Advisory Committee for MHRA database Research.

#### Linkage

The anonymised patient identifiers from GPRD, HES, and the Office of National Statistics (ONS) death register have been linked by a trusted third party using the NHS number, date of birth and gender [10]. As HES only covers English hospitals, any practices from Northern Island, Wales and Scotland were excluded. For this study we used the January 2011 download of GPRD GOLD data, in which 51.3% of GPRD primary care practices within England consented for their data to be linked.

### Defining upper gastrointestinal haemorrhage separately within primary care and secondary care data

#### Defining cases in the general practice research database

Primary care bleed events were defined in GPRD using Read codes that indicated a definite diagnosis or symptom of upper gastrointestinal bleed. Codes for unspecified gastrointestinal bleeding were also included to be consistent with previously published ICD 10 code lists [1,2,11], but they were excluded if they had a code for a lower gastrointestinal diagnosis or procedure. Primary care bleed events were excluded if the patient was under 18 years old, had temporary registration, had invalid date codes, was coded as elective or daycase, or occurred outside the observed and up to standard time period. The start of the observed and up to standard time period was defined as the latest of; the up to standard data collection date, 1st April 1997 (start of matching of GPRD and HES), or 3 months post current primary care registration (to avoid matching of prevalent events recorded during a new patient registration [12]). The end of this observed time period was defined by the earliest of; date of death, date of transfer out of practice, 31st August 2010 (end of matched HES data in current linkage) or the last collection date for the practice.

#### Defining cases in the hospital episodes statistics database

Secondary care bleed admissions were defined in HES using a published ICD 10 code list for upper gastrointestinal haemorrhage [11] and we further refined it by excluding unspecified gastrointestinal haemorrhage codes that also had either a lower gastrointestinal procedure or diagnosis coded [1,2]. Multiple admissions were included for each patient. Secondary care events were excluded if the patient was under 18 years old, had temporary registration in primary care, had invalid date codes, was coded as elective or daycase, or occurred outside the observed and up to standard time period as defined in the previous section for GPRD.

### Defining concurrent events in the linked databases

#### Defining time windows for concurrent codes in primary and secondary care

The timing of the coding of an event in primary and secondary care might differ due to communication delays. The standard within the NHS for hospital communications is that a discharge letter, with a minimum of the main discharge diagnosis and prescriptions, should be sent to the primary care doctor within 24 hours of discharge [13]. A time difference greater than 2 months was judged too long for delivery of discharge letters and its subsequent coding, and we therefore used 2 months as the cut off for associating separate events from the linked datasets. Other time windows, allowing for intermediate delays in primary care coding, were defined as less than 2 months, 1 month, 2 weeks, or 1 week, pre or post the event defined in either primary or secondary care. An event of upper gastrointestinal bleeding might have been coded first in either primary care prior to referral or in secondary care on the admission date. We therefore selected the earlier of the two dates as the index date for the 28 day case fatality analysis.

#### Defining acceptable concurrent codes in primary and secondary care

Upper gastrointestinal bleeding might not be identically coded in primary and secondary care because an upper gastrointestinal bleed code in one database could have a number of legitimate corresponding codes in the linked database: For example outcomes such as death or collapse, underlying diagnoses such as cancer, or procedures such as oesophagogastroduodenoscopy. To allow for this heterogeneity in coding, ‘probable’ and ‘possible’ groups of ICD 10 and Read codes were selected that could plausibly be coded following an upper gastrointestinal bleed. ‘Probable’ codes were defined as those specifying a likely symptom, cause, therapy, investigation or outcome of upper gastrointestinal haemorrhage. ‘Possible’ codes were defined as less specific codes that nevertheless indicated a non specific change in health state without indicating an alternative diagnosis to a gastrointestinal bleed (see Table 1 for categories of codes, 1=Most probable, 16=Less probable. The specific codes selected are listed in Additional file 1: Table S1 and Additional file 2: Table S2). This was based on the clinical judgement of the authors (2 consultant gastroenterologists and 1 trainee gastroenterologist).

**Table 1:** Categories of read or ICD 10 codes that might be associated with a hospital admission for upper gastrointestinal haemorrhage

**Category Order**	**Group name**	**Group definition**	**Probable or possible codes**
1	Upper GI bleed cause	Code for known upper GI bleed diagnosis or cause. e.g. ulcer, oesophagitis, NSAID or Aspirin use, cirrhosis, upper GI malignancy etc.	Probable
2	Upper GI bleed symptom	Symptoms indicating upper GI bleed e.g. melaena, haematemesis etc.	Probable
3	Upper GI endoscopy	Any upper GI endoscopy code (Not ERCP/EUS).	Probable
4	Death (any cause)	Any code associated with death.	Probable
5	Blood transfusion	Any code for blood transfusion or cross matching.	Probable
6	Upper GI procedure	Any code for an upper GI procedure plausible for managing a bleeding episode.	Probable
7	GI bleed symptom	Any general code for GI bleed (not specifically upper or lower).	Probable
8	Upper GI diagnosis	Any other code for an upper GI pathology that might be associated with an upper GI bleed.	Possible
9	Hospital	Any code for referral, admission or discharge to hospital in a general or related specialty.	Possible
10	Upper GI symptom	Any other code for symptoms of upper GI pathology e.g. vomiting.	Possible
11	GI symptom or diagnosis	Other GI diagnoses or non specific GI symptoms(e.g. pain) excluding lower GI symptoms.	Possible
12	Alcohol	Any code indicating alcohol consumption or complications.	Possible
13	Anaemia	Any code for anaemia excluding chronic deficiency anaemias and fatigue.	Possible
14	Coagulation	Any code indicating primary or secondary clotting abnormality, or use of anti coagulation therapy.	Possible
15	Collapse	Any code indicating collapse, fall or loss of consciousness.	Possible
16	Other codes	Other codes specifying a change in health state with no specific diagnosis	Possible

Listed in Order of how Probable a Code Category Would be Associated with an Upper Gastrointestinal Haemorrhage Admission (1=Most Probable).

### Classification of case definitions of upper gastrointestinal bleeding in linked primary and secondary care data

We defined four case definitions of differing specificity and assessed how this altered our study population in regard to occurrence and case fatality. All four case definitions required at least a specific upper gastrointestinal bleeding code from one database with or without a code from the linked dataset that was of differing specificity; from the broad and sensitive case definition 1 that requires no linked code, to the restrictive and specific case definition 4 requiring a specific bleeding code. For each case definition cases initially defined from each dataset (identified as (a) for HES and (b) for GPRD) were pooled and duplicates excluded. Duplicate events were identified as those that occurred within the 2 month time window we used for defining corresponding codes.

Definition 1-All secondary and primary care defined events

This broad and sensitive definition selected all possible cases of upper gastrointestinal bleeding from the linked data. All cases defined by a specific Read or ICD 10 bleed code in either database were combined and duplicate events were excluded.

**Definition 2a & 2b-Primary and secondary care events that had a concurrent ‘Probable’ or ‘Possible’ code in the linked dataset**.

This definition selected all cases of upper gastrointestinal bleeding that had either a supporting code (probable code) in the linked data or a less specific code (possible code) that did not contradict the bleeding diagnosis. Therefore for each upper gastrointestinal bleed defined in either dataset from definition 1, a specific bleed code, probable code or possible code was searched for in the linked dataset within the 2 month time window and selected in the hierarchical order of the categories listed in Table 1. Each primary care event was matched to only one hospital admission that was closest in time and vice versa.

Definition 3a & 3b-Primary and secondary care events that had a concurrent ‘Probable’ code in the linked database.

This definition selected all cases of upper gastrointestinal bleeding that had a code in the linked data that supported the diagnosis of bleeding. This required restricting the cases defined in 2a & 2b to only those with a more specific probable code in the linked dataset.

Definition 4-Primary and secondary care events with specific bleed codes in both GPRD and HES

To provide a very specific case definition only those with a specific upper gastrointestinal bleed code in both primary and secondary care datasets were selected.

### Analysis: Incidence and 28 day case fatality by case definition

The incidence was calculated per 100,000 person years; the denominator was the underlying number of person years contributed by patients registered in the GPRD and the numerator was calculated by pooling each of the case definitions from the GPRD and HES ((a) and (b) respectively for each of the definitions above).

Finally we assessed the effect of each of these case definitions on the results of our intended studies in linked primary and secondary care data. Within the general population registered to a linked GPRD primary care practice we calculated the numbers of cases identified by each case definition and the subsequent all cause 28 day case fatality. Dates of all deaths within 28 days following an upper gastrointestinal bleed admission date or primary care event date were ascertained using the linkage between the GPRD primary care practices and the UK Office for National Statistics death register.

## Results

### Defining upper gastrointestinal bleeding separately within primary care and secondary care data

Between 1st April 1997 and 30th August 2010 30,176 acute upper gastrointestinal bleed events were indentified in the linked primary care GPRD data by specific Read bleed codes (Table 2) and 26,964 acute upper gastrointestinal bleed admissions were identified in the linked secondary care HES data by specific ICD 10 bleed codes (Table 3). Combining these events and excluding duplicates defined 45,472 unique upper gastrointestinal bleed events, 26% with a specific code in both datasets, 34% with a code only in HES and 41% with a code only in GPRD. The proportion of all events defined by specific bleed codes from both databases varied by year between 22%-27% but there was no clear trend over time.

**Table 2:** The frequency of read codes used to define upper gastrointestinal cases in the general practice research database

**Read code description**	**Read code**	**Frequency**
Haematemesis	J680.00	10918
Melaena	J681.00	6957
GIB - Gastrointestinal bleeding	J68z.11	3464
Gastrointestinal haemorrhage	J68.00	1203
Coffee ground vomit	4A24.11	968
Mallory - Weiss tear	J108.00	755
Vomiting of blood	J680.11	494
Mallory-Weiss syndrome	J107.00	427
Upper gastrointestinal haemorrhage	J68z200	280
Oesophageal varices with bleeding	G850.00	211
C/O – melaena	19E4.12	160
Acute haemorrhagic gastritis	J150000	108
Blood in vomit – symptom	1994.11	104
Blood in stools altered	J681.13	80
Bleeding acute gastric ulcer	J110111	74
Bleeding chronic duodenal ulcer	J121111	61
Gastric haemorrhage NOS	J68z000	60
Gastrointestinal tract haemorrhage NOS	J68zz00	48
Gastrointestinal haemorrhage unspecified	J68z.00	33
Vomiting blood – fresh	1994.00	30
Vomiting blood - coffee ground	1995.00	30
Unspecified duodenal ulcer with haemorrhage	J12y100	25
Intestinal haemorrhage NOS	J68z100	23
Acute duodenal ulcer with haemorrhage	J120100	19
Altered blood in stools	J681.12	18
Melaena - O/E of faeces	4737.11	16
Acute gastric ulcer with haemorrhage	J110100	13
Faeces colour: tarry	4737.00	12
Vomit: coffee ground	4A24.00	12
Haemorrhage of oesophagus	J10y000	11
Aorto-duodenal fistula	G762000	7
Bleeding chronic gastric ulcer	761D500	6
Endoscopic injection haemostasis of duodenal ulcer	J111111	6
Oversew of blood vessel of duodenal ulcer	7627200	4
Gastrotomy and ligation of bleeding point of stomach	4A23.11	3
Blood in vomit O/E	7619100	3
Chronic duodenal ulcer with haemorrhage	J111100	3
Chronic gastric ulcer with haemorrhage	J121100	3
Acute duodenal ulcer with haemorrhage and perforation	4A23.00	2
Chronic peptic ulcer with haemorrhage	J11y100	2
Vomit: frank blood present	J120300	2
Unspecified gastric ulcer with haemorrhage	J131100	2
Unspec duodenal ulcer; unspec haemorrhage and/or perforation	J111300	1
Unspecified peptic ulcer with haemorrhage	J12y300	1
Chronic gastric ulcer with haemorrhage and perforation	J12yy00	1
Unspecified duodenal ulcer with haemorrhage and perforation	J13y100	1
Total		26661

**Table 3:** The frequency of ICD 10 codes used to define upper gastrointestinal cases in the hospital episodes statistics database

**ICD 10 description**	**ICD 10**	**Frequency**
Haematemesis	K920	9359
Melaena	K921	5802
Gastrointestinal haemorrhage unspecified	K922	4014
Duodenal ulcer chronic or unspecified with haemorrhage	K264	1333
Gastro-oesophageal laceration-haemorrhage syndrome	K226	1171
Gastric ulcer chronic or unspecified with haemorrhage	K254	1038
Duodenal ulcer acute with haemorrhage	K260	529
Acute haemorrhagic gastritis	K290	525
Oesophageal varices with bleeding	I850	517
Haemorrhage of oesophagus	K228	483
Gastric ulcer acute with haemorrhage	K250	399
Peptic ulcer chronic or unspecified with both haemorrhage and perforation	K274	88
Duodenal ulcer chronic or unspecified with haemorrhage	K266	81
Duodenal ulcer acute with both haemorrhage and perforation	K262	38
Gastric ulcer acute with chronic or unspecified with both haemorrhage and perforation	K256	31
Peptic ulcer acute with haemorrhage	K270	20
Gastric ulcer acute with both haemorrhage and perforation	K252	17
Gastrojejunal ulcer chronic or unspecified with haemorrhage	K284	14
Gastrojejunal ulcer acute with haemorrhage	K280	6
Peptic ulcer chronic or unspecified with haemorrhage	K276	3
Gastrojejunal ulcer chronic or unspecified with haemorrhage	K286	2
Gastrojejunal ulcer with both haemorrhage and perforation	K282	1
Total		25471

### Classification of case definitions of upper gastrointestinal bleeding in primary and secondary care

**Figure 1:**
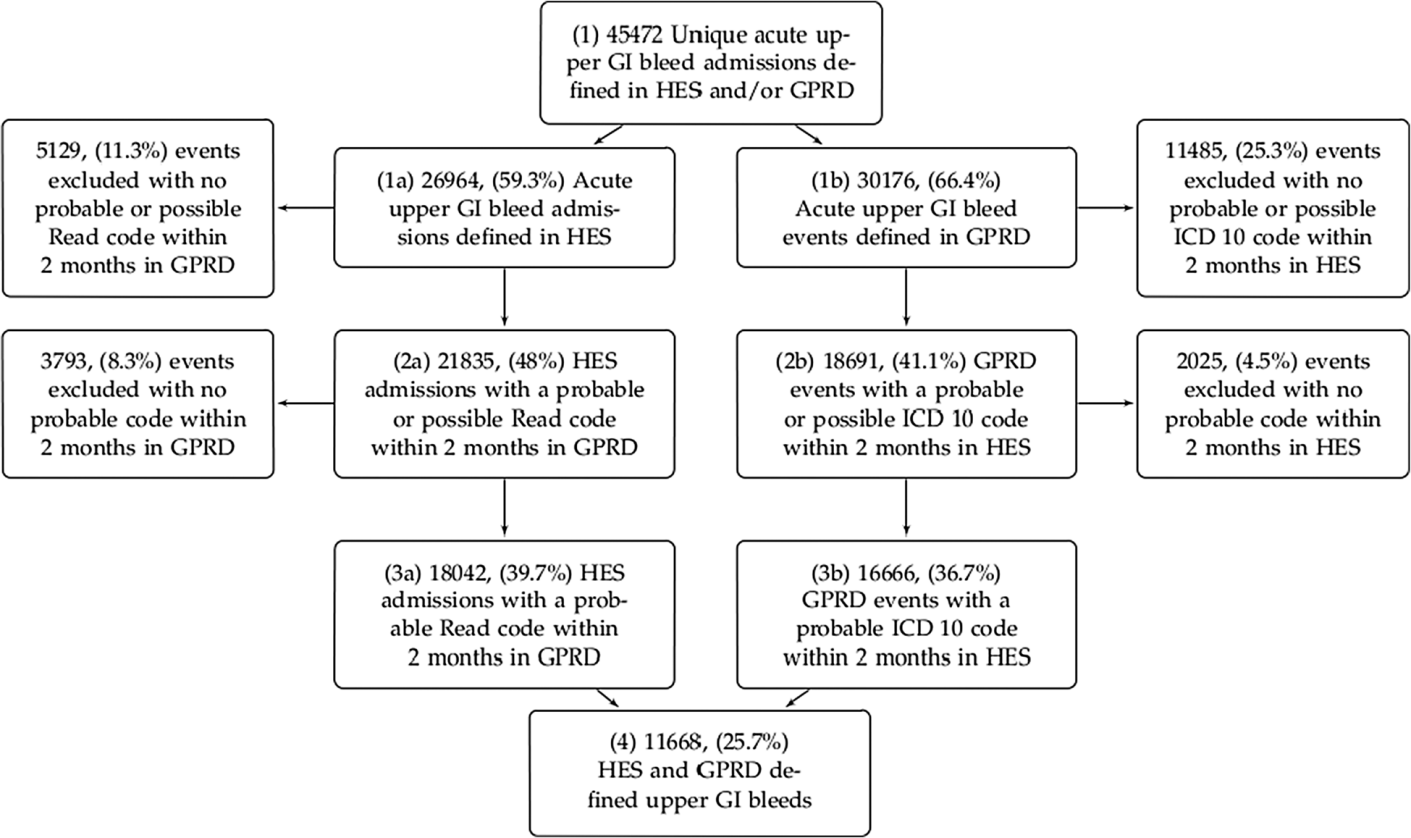
**Upper Gastrointestinal Bleed Events Defined in Linked HES and GPRD Data, England, 1997–2010.** The percentages shown are of the combined unique events in box 1. GPRD- General Practice Research Database; HES - Hospital Episodes Statistics; ICD 10 - International Classification of Diseases 10th Edition; GI - Gastrointestinal; READ- Read code.

The flow chart in Figure 1 shows the selection of adult upper gastrointestinal bleeding events for each of our four case definitions. The percentages given in the flow chart are of the 45,472 pooled unique events in box 1. Of the 26,964 secondary care defined bleeds in box 1a, 81% had a ‘Probable’ or ‘Possible’ code in primary care within 2 months (box 2a, Figure 1). By comparison 62% of the 30,176 primary care defined bleeds in box 1b had a ‘Probable’ or ‘Possible’ secondary care code within 2 months (box 2b, Figure 1). Further details of the timings of the closest ‘Possible’ or ‘Probable’ codes to the defining upper gastrointestinal bleed code date are shown in Tables 4 &5.

**Table 4:** Timing of probable or possible primary care events to secondary care defined upper gastrointestinal bleed admissions

**Time difference between defining hospital event and closest probable or possible primary care event**	**Frequency**	**Percentage**	**Cumulative percentage**
Exact match	17,032	63.17	63.17
1 day prior or 1 week post	2,010	7.45	70.62
2 weeks pre or post event	707	2.62	73.24
1 month pre or post event	997	3.70	76.94
2 months pre or post event	1,089	4.04	80.98
> 2 months or no associated code	5,129	19.02	100.00
Total	26,964	100.00	

**Table 5:** Timing of probable or possible secondary care events to primary care defined upper gastrointestinal bleed events

**Time difference between defining primary care event and closest probable or possible secondary care event**	**Frequency**	**Percentage**	**Cumulative percentage**
Exact match	15,689	51.99	52.99
1 day prior or 1 week post	1,348	4.47	56.46
2 weeks pre or post event	469	1.55	58.01
1 month pre or post event	612	2.03	60.04
2 months pre or post event	573	1.90	61.94
> 2 months or no associated code	11,485	38.06	100.00
Total	30,176	100.00	

### Incidence and 28 day all cause case fatality by case definition

Incidence was calculated for each of the pooled case definitions and these are shown in Table 6. Incidence followed a similar pattern to the crude numbers in Figure 1.

**Table 6:** Pooled incidence for each case definition per 100,000 person years

**Pooled case definitions**	**Incidence per 100,000**	**95% confidence interval**
1a & 1b	224	(222–226)
2a & 2b	136	(134–138)
3a & 3b	114	(112–115)
4a & 4b	58	(57–59)

(Pooled between GPRD defined cases and HES defined cases).

**Figure 2:**
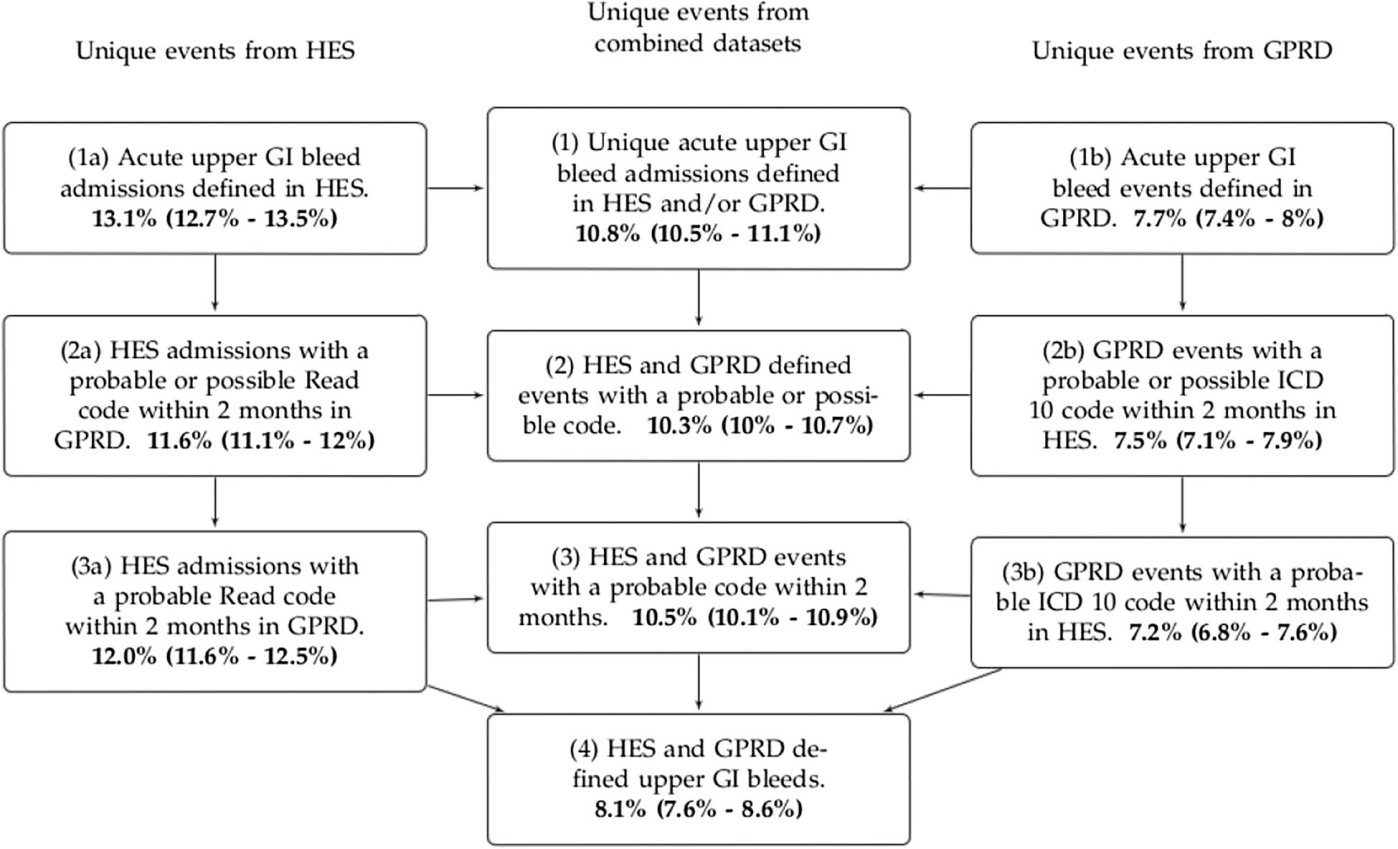
**28 Day Case Fatality of Upper Gastrointestinal Bleed Events in linked HES and GPRD Data, England, 1997–2010.** (95% Confidence Intervals shown in brackets). GPRD- General Practice Research Database; HES - Hospital Episodes Statistics; ICD 10 - International Classification of Diseases 10th Edition; GI - Gastrointestinal; READ- Read code; PATID - Patient Identifier in GPRD.

4,916 deaths were identified within 28 days of a bleed event using the linked ONS death register. 28 day mortality was calculated for each of the different case selections (Figure 2). Secondary care defined events had almost twice the case fatality of primary care defined events; 13.1% compared to 7.7% (box (1a) versus box (1b) in Figure 2).

Overall 28 day case fatality for all events defined in either GPRD or HES was 10.8% (box (1)). Selecting events from the combined datasets with an associated ‘Probable’ or ‘Possible’ code reduced the 28 day case fatality slightly (10.3%, box (2)) in Figure 2). Restricting the events to only those with a ‘Probable’ code had minimal affect on case fatality (10.5%, box (3)) in Figure 2). However further restricting events to those that were defined by specific upper gastrointestinal haemorrhage codes in both primary and secondary care was associated with a much lower case fatality (8.1%, box (4) compared to 10.5%, box (3)) in Figure 2).

## Discussion

This study assessed the effect of different case definitions of upper gastrointestinal bleeding on its measured incidence and mortality in linked primary and secondary care data. We used the record linkage between the world’s largest hospital admissions databases and one of the most commonly used primary care databases from the UK. Cases defined only in hospital data were at twice the risk of dying compared to those defined only in primary care data. Furthermore we found that the most specific case definition, which restricted to specific bleed codes from both datasets, excluded severe cases and resulted in a lower 28 day case fatality. In contrast the more sensitive case definitions, using the broader possible or probable code lists, retained the more severe cases and did not reduce the overall case fatality. Therefore studies that are too restrictive in their case definitions will fail to capture the full heterogeneity of coding that follows complex or severe clinical events, and potentially introduce selection bias.

Reassuringly we found that 81% of upper gastrointestinal bleed events coded in secondary care had a probable or possible record in primary care within two months. However less than two thirds of upper gastrointestinal bleed events coded in primary care were associated with a hospital admission within the same time window. Therefore primary care could be recording sub acute bleeding episodes or symptoms that were historical at the time of the consultation, and therefore these patients did not require acute hospital admission. This is supported by the lower 28 day case fatality in events defined in GPRD alone compared to those also defined in HES. Coded bleeding events with no hospital admission were potentially interesting to investigate but were not representative of the acute bleeds described in studies of upper gastrointestinal bleeding management [14,15].

One of the limitations of this study is that the anonymisation of HES data prevents the validation of individual records against the original clinical chart records. Although this potentially leaves the database susceptible to accusations of poor coding quality [16,17], the most recent government audit of selected samples of UK hospital data confirmed accuracy approaching 90% [18]. Other comparisons have reported similar rates of procedure coding in HES compared to specialist databases [19,20] and the incidence of peptic ulcer haemorrhage in HES (1992–1995) was to comparable to the 1993 regional BSG audit (32 v 29 per 100,000 per year respectively). Furthermore the recent prospective national audit in the UK recorded reassuringly similar numbers for upper gastrointestinal bleed hospital admissions and procedures as were recorded in HES over the same period [3] and the outcome measure of 28 day case fatality following admission was similar to previous national audits [21,22] and the whole of the HES database [2].

In the GPRD the positive predictive value of an upper gastrointestinal bleed coding was 99% using anonymised chart review [4]. However, we believe that the linkage of GPRD and HES, and the comparison presented in this paper, provides a more comprehensive and less biased assessment of the validity of the coding from both datasets than from small sample validation, as all potential cases were assessed and compared. Furthermore this linkage allows the comparison of coding by primary care doctors against the coding by trained hospital personnel using secondary care doctors’ notes, thereby supporting any resulting case definitions from two separate and independent data sources.

There have been other databases linked for a range of purposes. However many, like those based on Health Maintenance Organisations, are limited by incomplete or selected population coverage because they are not based on a comprehensive population based primary health care system [23,24]. Scandinavian linked databases are the most established [25,26], but they do not have the richness of the data collection in primary care that the GPRD records, such as lifestyle factors, practice and individual socioeconomic status, occupation status, diagnoses, procedures, health promotion, and referrals. Prior to the linkage of HES and GPRD it was only possible to compare these databases using aggregate measures [27], and the new record level linkage avoids these ecological biases. The use of both primary and secondary care has previously been shown to be beneficial in defining chronic diseases such as diabetes [28,29], and using only primary care data reduced the positive predictive value for acute events [30]. Our study supports this finding for the acute event of upper gastrointestinal haemorrhage, and we propose that this issue can be addressed and improved upon by the use of linked hospital data.

We initially began this investigation to develop specific case definitions that minimise misclassification bias on effect estimates when testing aetiological hypotheses [31]. To achieve this we now intend to use a specific upper gastrointestinal bleed code in one dataset with a probable or specific code in the linked dataset (box 3a & 3b, Figure 1). This will select the most plausible cases of acute upper gastrointestinal haemorrhage without excluding severe cases (box 3, Figure 2). In contrast to an aetiological study, studies that estimate incidence require a broader and more sensitive case definition to be sure of capturing all cases of the disease in question [31]. For incidence studies we therefore propose using all hospital defined cases with the addition of primary care defined cases that have a plausibly coded hospital admission (Figure 1, box (1a) and box (2b)). A sensitivity analysis that also included the events defined only in primary care (box 1b) would then provide an upper estimate of bleed events in the population. Equally for mortality studies a similar broad and sensitive definition might be preferable.

## Conclusions

In this study we have been able to establish case definitions for upper gastrointestinal bleeding based on linked primary and secondary care data, and shown that linked data can be used to avoid excluding severe events. We have shown that hospital data was invaluable in accurately identifying acute bleeding events that were severe enough to require hospital admission, and the recent linkage with primary care introduced a wealth of long term diagnosis data and prescription data for the researcher. In addition there was a close match in timing in primary and secondary care between events coded for upper gastrointestinal haemorrhage. Finally we have shown that the choice of definition in linked data has a clear effect on the mortality of the chosen population. Our methods may not be generalisable to the definition of chronic diseases in linked databases, as chronic disease diagnoses are usually made in outpatient clinics and primary care. However we believe our findings are likely to be generalisable and relevant to other acute severe events, such as myocardial infarction or venous thromboembolism that are investigated, diagnosed, and managed during an acute hospital admission.

## Competing interests

The only competing interest is that Tim Card is married to an employee of AstraZeneca. Otherwise there are no potential competing interests to declare.

## Authors’ contributions

All authors were involved in the study concept, design, interpretation of results, and editing the manuscript. CC performed the data analysis and initial draft manuscript. All authors read and approved the final manuscript.

## Pre-publication history

The pre-publication history for this paper can be accessed here:

http://www.biomedcentral.com/1472-6963/12/392/prepub

## Supplementary Material

Additional file 1 **Table S1.** Word document file containing a table of the ICD 10 supporting codes in each category and their frequency in this study. Click here for file

Additional file 2 **Table S2.** Word document file containing a table of the Read supporting codes in each category and their frequency in this study. Click here for file
