# Supplementary material for: Defining upper gastrointestinal bleeding from linked primary and secondary care data and the effect on occurrence and 28 day mortality
Source: BMC Health Serv Res. 2012 Nov 13;12:392. doi: 10.1186/1472-6963-12-392 (PMC3531298; doi:10.1186/1472-6963-12-392)
Supplement: Additional file 1 — Table S1. Word document file containing a table of the ICD 10 supporting codes in each category and their frequency in this study. [file 1472-6963-12-392-S1.docx]

Supplementary table A

| Category of supporting ICD 10 codes in Hospital Episodes Statistics for cases defined by a specific Read code in the General Practice Research Database | Frequency |
| --- | --- |
| Upper GI bleed symptom  D62 , K922 , K9229 | 5595 |
| Upper GI bleed cause  C150 , C152 , C153 , C154 , C155 , C158 , C159 , C160 , C161 , C162 , C163 C164 , C165 , C166 , C168 , C169 , C170 , D001 , D130 , D131 , D132 , D139 D371 , D379 , I81 , I850 , I864 , I982 , K20 , K210 , K221 , K223 , K226 K250 , K252 , K254 , K255 , K256 , K260 , K261 , K262 , K264 , K265 , K266 K270 , K274 , K275 , K280 , K284 , K285 , K290 , K291 , K292 , K293 , K294 K295 , K296 , K297 , K298 , K299 , K317 , K500 , K508 , K509 , K766 , K767 S363 , T390 , T393 , Y451 , Y453 , Z850 | 5521 |
| Upper GI endoscopy  Y604 , Y614 | 3923 |
| GI bleed symptom  K920 , K921 | 884 |
| GI symptom or diagnosis  B161 , B169 , B171 , B178 , B180 , B181 , B182 , B188 , B189 , C171 , C178 C220 , C229 , C250 , C258 , C259 , C269 , C482 , C762 , C787 , C788 , C798 C80 , C97 , D099 , D133 , D134 , D367 , D369 , D372 , D377 , E164 , I780 I820 , K550 , K551 , K558 , K559 , K561 , K562 , K563 , K564 , K565 , K566 K630 , K631 , K632 , K633 , K638 , K639 , K710 , K711 , K713 , K716 , K718 K719 , K720 , K721 , K729 , K730 , K732 , K738 , K739 , K740 , K741 , K743 K744 , K745 , K746 , K750 , K751 , K753 , K754 , K758 , K759 , K760 , K761 K762 , K763 , K765 , K768 , K769 , K770 , K860 , K910 , K911 , K912 , K913 K918 , K928 , K929 , K938 , M352 , O266 , Q433 , Q438 , Q439 , Q446 , Q447 Q458 , R100 , R101 , R102 , R103 , R104 , R160 , R162 , R17 , R18 , R190 R193 , R198 , R850 , R855 , R857 , R859 , R890 , R895 , R897 , R899 , R933 R945 , R948 , T478 , T479 , Y538 , Y539 , Z221 , Z225 , Z400 , Z434 , Z871 Z8713 , Z904 | 535 |
|  |  |
| Upper GI diagnosis  K219 , K220 , K222 , K224 , K225 , K228 , K229 , K230 , K231 , K238 , K30 K310 , K311 , K312 , K313 , K314 , K315 , K316 , K318 , K319 , K440 , K441 K449 , K450 , K451 , K458 , K460 , K469 , Q391 , Q393 , Q394 , Q396 , Q401 Q402 , Q403 , S368 , T181 , T182 , T183 , T189 , Z903 | 453 |
| Upper GI symptom  O210 , O211 , O218 , O219 , R11 , R12 , R13 | 347 |
| General care  W000 , W002 , W004 , W008 , W009 , W010 , W0109 , W011 , W0114 , W0119 , W012 W0129 , W013 , W014 , W0149 , W015 , W016 , W018 , W0188 , W019 , W0199 , W021 W023 , W024 , W029 , W030 , W031 , W033 , W034 , W035 , W038 , W039 , W040 W042 , W044 , W049 , W050 , W051 , W052 , W054 , W058 , W059 , W060 , W0609 W061 , W0619 , W062 , W0629 , W068 , W069 , W070 , W0709 , W071 , W072 , W0729 W074 , W075 , W079 , W080 , W081 , W082 , W088 , W089 , W090 , W091 , W098 W099 , W100 , W1009 , W101 , W102 , W103 , W104 , W105 , W108 , W109 , W110 W115 , W116 , W118 , W119 , W125 , W129 , W130 , W131 , W132 , W134 , W138 W139 , W140 , W148 , W149 , W160 , W170 , W171 , W172 , W174 , W175 , W177 W178 , W179 , W1799 , W180 , W1809 , W181 , W1819 , W182 , W1829 , W183 , W184 W185 , W186 , W188 , W189 , W190 , W1909 , W191 , W1919 , W192 , W1923 , W1929 W193 , W194 , W1949 , W195 , W198 , W199 , W1999 , W200 , W205 , W206 , W209 W213 , W220 , W221 , W222 , W223 , W224 , W225 , W226 , W227 , W228 , W229 W230 , W231 , W232 , W234 , W236 , W238 , W239 , W241 , W250 , W2508 , W252 W254 , W255 , W256 , W258 , W259 , W260 , W268 , W269 , W270 , W272 , W274 W276 , W279 , W280 , W289 , W290 , W298 , W299 , W310 , W312 , W315 , W316 W3162 , W318 , W319 , W344 , W349 , W4059 , W406 , W440 , W441 , W442 , W449 W4499 , W450 , W451 , W455 , W458 , W459 , W490 , W492 , W496 , W499 , W500 W503 , W508 , W509 , W5098 , W510 , W511 , W513 , W514 , W518 , W519 , W540 W544 , W548 , W549 , W550 , W558 , W559 , W570 , W573 , W579 , W5799 , W599 W600 , W609 , W6099 , W640 , W642 , W649 , W699 , W740 , W748 , W780 , W781 W782 , W789 , W790 , W791 , W792 , W799 , W7999 , W800 , W802 , W809 , W839 W840 , W842 , W849 , W850 , W877 , W882 , W909 , W948 , X000 , X011 , X020 X039 , X049 , X060 , X069 , X080 , X089 , X090 , X099 , X100 , X102 , X109 X110 , X120 , X121 , X129 , X149 , X150 , X159 , X160 , X162 , X169 , X175 X186 , X190 , X195 , X199 , X209 , X219 , X239 , X258 , X292 , X310 , X314 X318 , X319 , X329 , X332 , X360 , X391 , X394 , X398 , X399 , X400 , X401 X402 , X408 , X409 , X4099 , X410 , X411 , X412 , X418 , X419 , X420 , X421 X422 , X424 , X428 , X429 , X430 , X439 , X440 , X441 , X442 , X448 , X449 X450 , X458 , X459 , X469 , X470 , X476 , X478 , X479 , X490 , X491 , X498 X499 , X4999 , X500 , X5008 , X5009 , X501 , X502 , X503 , X504 , X505 , X506 X508 , X5089 , X509 , X5099 , X519 , X530 , X539 , X580 , X581 , X582 , X588 X589 , X5899 , X590 , X5909 , X591 , X592 , X593 , X594 , X595 , X596 , X598 X5989 , X599 , X5999 , X600 , X6009 , X601 , X602 , X604 , X608 , X609 , X6099 X610 , X6109 , X611 , X612 , X614 , X615 , X618 , X619 , X6199 , X620 , X622 X624 , X628 , X629 , X6299 , X630 , X638 , X639 , X640 , X642 , X648 , X649 X6499 , X650 , X651 , X652 , X654 , X658 , X659 , X6599 , X660 , X669 , X670 X678 , X680 , X689 , X690 , X691 , X698 , X699 , X700 , X701 , X702 , X709 X718 , X749 , X760 , X770 , X771 , X780 , X781 , X782 , X788 , X789 , X790 X791 , X792 , X795 , X799 , X800 , X804 , X808 , X818 , X824 , X830 , X831 X832 , X834 , X838 , X839 , X840 , X841 , X842 , X849 , X853 , X866 , X870 X900 , X932 , X950 , X979 , X990 , X992 , X994 , X999 , Y000 , Y001 , Y004 Y008 , Y009 , Y010 , Y040 , Y041 , Y042 , Y044 , Y045 , Y048 , Y049 , Y0499 Y053 , Y070 , Y079 , Y080 , Y084 , Y088 , Y089 , Y090 , Y094 , Y095 , Y098 Y099 , Y100 , Y109 , Y110 , Y112 , Y119 , Y120 , Y129 , Y139 , Y140 , Y149 Y150 , Y159 , Y179 , Y190 , Y199 , Y218 , Y219 , Y249 , Y280 , Y281 , Y289 Y292 , Y294 , Y300 , Y304 , Y309 , Y331 , Y332 , Y340 , Y341 , Y342 , Y349 Y3499 , Y95 , Z000 , Z005 , Z006 , Z008 , Z013 , Z018 , Z0180 , Z019 , Z031 Z036 , Z038 , Z039 , Z040 , Z043 , Z048 , Z049 , Z080 , Z081 , Z082 , Z087 Z088 , Z089 , Z090 , Z092 , Z097 , Z098 , Z099 , Z120 , Z121 , Z128 , Z129 Z132 , Z138 , Z1380 , Z480 , Z488 , Z489 , Z508 , Z515 , Z518 , Z519 , Z530 Z531 , Z532 , Z538 , Z539 , Z547 , Z548 , Z549 , Z593 Z728 , Z729 , Z750 Z751 , Z752 , Z753 , Z758 , Z759 , Z764 , Z858 Z878 , Z910 , Z911 , Z922 Z924 , Z929 | 329 |
| Alcohol  E244 , F100 , F101 , F102 , F103 , F104 , F105 , F108 , F109 , G312 , G621 G721 , I426 , K700 , K701 , K702 , K703 , K704 , K709 , R780 , T510 , T511 T519 , Y905 , Y906 , Y908 , Y910 , Y911 , Y912 , Y913 , Y919 , Z502 , Z714 Z721 | 268 |
| Anaemia  D649 | 176 |
| Upper GI procedure  Z431 , Z931 | 140 |
| General symptom or diagnosis  B378 , B379 , B948 , C768 , C772 , C778 , C779 , C786 , D479 , D484 , D487 D489 , D630 , E519 , G92 , G934 , R231 , R402 , R520 , R529 , R53 , R54 R58 , R688 , R69 , T394 , T398 , T399 , T475 , T485 , T490 , T509 , T658 T659 , T788 , T789 , Y430 , Y431 , Y433 , Y454 , Y458 , Y459 , Y560 , Y578 Y579 , Z511 , Z514 , Z859 , Z860 | 137 |
| Collapse  E86 , I950 , I951 , I952 , I958 , I959 , R031 , R42 , R55 , R570 , R571 R578 , T794 , T795 , Z990 , Z991 , Z998 , Z999 | 105 |
| General procedure  T412 , T801 , T802 , T808 , T809 , T810 , T811 , T812 , T813 , T814 , T815 T816 , T817 , T818 , T819 , T855 , T864 , T868 , T869 , T884 , T885 , T886 T887 , T888 , T889 , T915 , T96 , T981 , T983 , Y482 , Y484 , Y600 , Y606 Y610 , Y618 , Y638 , Y649 , Y652 , Y654 , Y658 , Y66 , Y701 , Y703 , Y710 Y711 , Y712 , Y730 , Y732 , Y733 , Y738 , Y741 , Y772 , Y773 , Y778 , Y780 Y792 , Y793 , Y801 , Y808 , Y812 , Y822 , Y828 , Y830 , Y831 , Y832 , Y833 Y834 , Y836 , Y838 , Y839 , Y842 , Y845 , Y847 , Y848 , Y849 , Y880 , Y881 Y882 , Y883 , Y899 , Z540 , Z948 , Z988 | 67 |
| Coagulation  D65 , D683 , D684 , D688 , D689 , D698 , D699 , E561 , O723 , T455 , T456, Y442 , Y443 , Y444 , Y445 , Z921 | 63 |
| Nutrition  R630 , R634 , R638 , R64 , Z594 | 26 |
| Confusion  R401 , R410 | 17 |
| Death  I460 , I469 , R092 , R960 , R99 | 16 |
| Blood transfusion  Y446 , Y449 , Z513 | 3 |
| GI procedure  Z934 , Z944 , Z980 | 2 |
