# Supplementary material for: Defining upper gastrointestinal bleeding from linked primary and secondary care data and the effect on occurrence and 28 day mortality
Source: BMC Health Serv Res. 2012 Nov 13;12:392. doi: 10.1186/1472-6963-12-392 (PMC3531298; doi:10.1186/1472-6963-12-392)
Supplement: Additional file 2 — Table S2. Word document file containing a table of the Read supporting codes in each category and their frequency in this study. [file 1472-6963-12-392-S2.docx]

Supplementary table B

| Category of supporting Read code in the General Practice Research Database for cases defined in Hospital Episodes Statistics | Frequency |
| --- | --- |
| Upper GI bleed symptom  14C8.00, 14C9.00, 14CD.00, 14CD.11, 1994 1994.11, 1995, 19E4.00, 19E4.11, 19E4.12, 4736 4737, 4737.11, 4A23.00, 4A23.11, 4A24.00 4A24.11, J680.00, J680.11, J681.00, J681.12 J681.13, J68z000 J68z200 | 7527 |
| Upper GI bleed cause  14C1.00, 14C1.11, 14C1.12, 14C5.00, 14C6.00 14CB.00, 1675, 1675.11, 1675.12, 1956, 1J0D.00 2274, 2274.11, 67I8.00, 7609300 760C.00 760C000, 760C100, 760C300, 760C400, 760C500, 760C600, 760C700, 760Cy00, 760Cz00, 760F.00, 760F100 760F400, 760H000, 761D.00, 761D.11, 761D000 761D100, 761D200, 761D300, 761D400, 761D500 761D600, 761D700, 761D800, 761Dy00, 761Dz00 761J.00, 761J.11, 761J000, 761J100, 761J111, 761Jy00 761Jz00, 761K.00, 761K000, 761M.00, 761M000 7624000, 7624011, 7625000, 7627, 7627000 7627100, 7627200, 8Hn9.00, A074313 B1...11, B10..00, B10z.00, B10z.11, B11..00, B11..11, B110.00, B110000, B110100, B110111, B110z00, B111.00, B111000, B111100, B111z00, B112.00, B113.00, B114.00, B115.00, B116.00, B117.00, B118.00, B119.00, B11y.00, B11y000, B11y100, B11yz00, B11z.00, B12..00, B120.00, B121.00, B574.00, B574000, B574z00, B70X.00, B71..00, B710.00, B710.11, B710100, B710300, B710z00, B711.00, B711.11, B711000, B711100, B711200, B711300, B711400, B711z00, B712.00, B712000, B712011, B712z00, C310400, G762000, G81..00, G85..11, G85..12, G850.00, G851.00, G852.00, G852000, G852100, G852200, G852300, G852z00, G857.00, G858.00, J101.00, J101100, J101112, J101113, J101114, J101115, J101200, J101300, J101400, J101500, J101600, J101611, J101y00, J101z00, J102.00, J102000, J102100, J102200, J102300, J102400, J102500, J102z00, J103.00, J103.11, J103.12, J103400, J103z00, J104.00, J107.00, J108.00, J10y000, J10y300, J10y400, J10y411, J10y412, J11..00, J11..11, J11..12, J110.00, J110000, J110100, J110111, J110200, J110300, J110y00, J110z00, J111.00, J111000, J111100, J111111, J111200, J111211, J111300, J111400, J111y00, J111z00, J112.00, J112z00, J113.00, J113z00, J11y.00, J11y000, J11y100, J11y200, J11y400, J11yy00, J11yz00, J11z.00, J11z.11, J11z.12, J12..00, J120.00, J120000, J120100, J120200, J120300, J120400, J120y00, J120z00, J121.00, J121000, J121100, J121111, J121200, J121211, J121300, J121400, J121y00, J121z00, J122.00, J123.00, J124.00, J125.00, J126.00, J12y.00, J12y000, J12y100, J12y200, J12y300, J12y400, J12yy00, J12yz00, J12z.00, J13..00, J13..11, J130.00, J130000, J130100, J130200, J130300, J130y00, J130z00, J131.00, J131000, J131100, J131200, J131400, J131y00, J131z00, J13y.00, J13y000, J13y100, J13y200, J13y300, J13y400, J13yz00, J13z.00, J14..00, J14..11, J14..12, J14..13, J14..14, J14..15, J140.00, J140100, J140z00, J141.00, J14y.00, J14y100, J14y200, J14yz00, J14z.00, J15..00, J150.00, J150000, J151.00, J151000, J151100, J151200, J151z00, J152.00, J153.00, J154.00, J154000, J154100, J154200, J154300, J154400, J154z00, J155.00, J156.00, J157.00, J15z.00, J17y800, J17y900, J40..11, J400000, J431000, J4z0.00, J502000, J612.00, J612.11, J612.12, J612000, J615.11, J615100, J615300, J615400, J615500, J615600, J615700, J615800, J615812, J615C00, J615D00, J615H00, J615y00, J615z00, J615z11, J615z12, J615z13, J61y300, J622.00, J622.11, J623.00, J624.00, Jyu1200, Jyu1300, Jyu4000, R024.00, R024111, R095.00, TJ53.00, TJ53.11, TJ56.11, U605100, U605111, U605112, U605200, U605211, U605212, U605213, U605214, U605215, U605216, U605300, U605311, U605312, U605313, U605314, U605315 | 3722 |
| GI bleed symptom  14CA.00, 14CA.11, 25T0.00, J68..00, J68z.00, J68z.11, J68z100, J68zz00 | 2335 |
| Upper GI endoscopy  316C.00, 36...00, 361..00, 3611, 3612, 3613, 3614, 3614000, 3615, 3615000, 361Z.00, 36Z..00, 4, JO..00, 4, JO0.00, 760D.00, 760D000, 760D100, 760D200, 760D300, 760D311, 760D313, 760D400, 760D500, 760D600, 760D700, 760Dy00, 760Dz00, 760E.00, 760E.11, 760E000, 760E100, 760E200, 760E300, 760Ey00, 760Ez00, 760Ez11, 760F300, 760G.00, 760G.11, 760G000, 760G100, 760G200, 760G300, 760G311, 760G400, 760Gy00, 760H.00, 760H100, 760Hy00, 761E.00, 761E.11, 761E000, 761E100, 761E200, 761E211, 761E300, 761E500, 761E600, 761E700, 761E800, 761Ey00, 761Ez00, 761F.00, 761F.11, 761F000, 761F100, 761F200, 761F300, 761F400, 761F500, 761F700, 761Fy00, 761Fz00, 761Fz11, 761Fz12, 761G200, 761G211, 761G400, 761L.00, 761L000, 761Ly00, 761Lz00, 761y.00, 761z.00, 7624100, 7624200, 7624y00, 7624z00, 7625, 7625.11, 7625y00, 7625z00, 7625z11 | 2240 |
| Death  22J..00, 22J..11, 22J..12, 22J..13, 22J..14, 22J1.00, 22J2.00, 22J3.00, 22J4.00, 22J5.00, 22J6.00, 22J7.00, 22JZ.00, 4K9..00, 4K91.00, 4K92.00, 4K94.00, 4K95.00, 4K96.00, 4K9Z.00, 8HG..00, 8HG..11, 94...00, 94...11, 941..00, 9411, 9412, 9413, 9414, 941Z.00, 942..00, 943..00, 9431, 9432, 9433, 943Z.00, 944..00, 9441, 9442, 9443, 944Z.00, 945..00, 9451, 9452, 9453, 9454, 945Z.00, 946..00, 947..00, 947..11, 9471, 9472, 9473, 947Z.00, 948..00, 948..11, 9481, 9482, 9483, 9484, 948Z.00, 949..00, 949..11, 949..12, 949..13, 949..14, 9491, 9492, 9493, 9494, 9495, 9496, 9497, 9498, 9499, 949A.00, 949B.00, 949C.00, 949Z.00, 94A..00, 94A..11, 94B..00, 94B..11, 94C..00, 94C0.00, 94D..00, 94E..00, 94F..00, 94Z..00, 94Z0.00, 94Z1.00, 94Z2.00, 94Z3.00, 94Z4.00, 94Z5.00, R2...12, R21..00, R210.00, R210000, R210100, R210200, R210z00, R211.00, R212.00, R212000, R212100, R212z00, R213.00, R213000, R213100, R213z00, R21z.00, R2y..00, R2yz.00, R2z..00 | 1305 |
| Upper GI symptom  194..00, 194..11, 1942, 1943, 1944, 1944.11, 194Z.00, 1952, 1952.11, 1954, 1955, 1955.11, 1957, 1958, 1972, 198..00, 198..11, 198..12, 1982, 1983, 1984, 198Z.00, 199..00, 199..11, 199..12, 199..14, 1992, 1992.11, 1992.12, 1993, 1996, 1997, 1998, 199Z.00, 19FZ.11, 4A25.11, 4A26.11, 4A27.00, 4A2A.11, 4A2Z.00, 4A3..00, 4A4..00, 4, A4..11, 4, A42.00, 4, A4Z.00, 4, A5..00, 4, A5..11, 4, A51.00, 4, A5Z.00, 4, A6..00, 4, AZ..00, 4, JD7.00, 4JN1.00, 4JS4.00, 7N3..00, 7N30.00, 7N30000, 7N30100, 7N30200, 7N30300, 7N30700, 7N30z00, 7N35000, J101111, J10y500, J10yz00, J10z.00, J162.00, J162.11, J162000, J162100, J162z00, J16y.00, J16y000, J16y100, J16y200, J16y211, J16y300, J16y400, J16y411, J16y500, J16y700, J16y800, J16y900, J16yz00, J16z.00, J16z100, J17..00, R070.00, R070000, R070100, R070200, R070300, R070400, R070z00, R070z11, R070z12, R071.00, R071000, R071z00, R072.00, R072000, R072z00, R07A.00 | 835 |
| Anaemia  145..11, 1674, 1674.11, 2272, 2272.11, 2272.12, 2C2..11, 421B.00, 4222, 423..00, 423..11, 4234, 4235, 4243, 4254, 4255, 4256, 426..00, 4262, 4263, 4266, 4267, 426Z.00, 42E8.00, 42R4200, 42X..00, 42X0.00, 42X2.00, 42bC.00, D211.00, D211.11, D21y.00, D21yy00, D21yz00, D21z.00, D2y..00, D2z..00, R026000, R026011 | 722 |
| General symptom or diagnosis  1....00, 13C6.00, 13C6.11, 13CA.00, 13CC.00, 142..12, 142..13, 14O..00, 14Z..00, 16...00, 16...11, 16...12, 16...13, 16E..00, 16E..11, 16E..12, 16E0.00, 16G..00, 16Z..00, 16Z3.00, 16Z7.00, 16Z8.00, 16Z9.00, 16ZZ.00, 1828, 1829, 182Z.00, 1D...00, 1, D1..00, 1, D13.00, 1D13.11, 1D13.12, 1D18.00, 1D1Z.00, 1J...00, 1M...00, 1O0..00, 1W...00, 1Y...00, 1Z...00, 1Z0..00, 1Z00.00, 1Z01.00, 2....00, 2....11, 2....12, 21...00, 211..00, 212..00, 2121, 2122, 2123, 2124, 2125, 2125.11, 2126, 2126.11, 2126.12, 2126.13, 2126.14, 2127, 2127.11, 2128, 2128.11, 2129, 212A.00, 212A.11, 212B.00, 212C.00, 212D.00, 212E.00, 212F.00, 212Z.00, 21Z..00, 22...00, 221..00, 2211, 2212, 2213, 2214, 2215, 221Z.00, 222..00, 2221, 2222, 2224, 2229, 2229.11, 2229.12, 2229.13, 222F.00, 222G.00, 222M.00, 223..00, 2231, 2252, 2253, 2271, 66W..00, 7N22000, 87...00, 87...11, 871..00, 8711, 8712, 8713, 8713.11, 871Z.00, 872..00, 872..11, 872..12, 8721, 8722, 8723, 8724, 872Z.00, 873..00, 8731, 8732, 8733, 8733000, 8733100, 8734, 8735, 873Z.00, C19..00, G8y0.00, R....00, R....11, R....12, R0...00, R00..00, R00z200, R00z211, R00zB00, R07..00, R070111, R073300, R073400, R2...00, R2...11, R2...13, R2...14, R200.00, R200.11, R201.00, R2y4.00, R2y4000, R2y4z00, R2yy.00, ZQ1..00, ZQ32.00 | 494 |
| Coagulation  1455.11, 1456, 14P1.00, 16B..00, 16B2.00, 16B3.00, 1928, 4130, 4224, 42Q..12, 42Q..13, 42Q2.00, 42Q3.00, 42Q4.00, 42Q5.00, 42Q5000, 42Q6.00, 42Q7.00, 42Q8.00, 42Q8100, 42QE.00, 42QE100, 42QV.00, 42QW.00, 42QX.00, 42QZ.00, 42Qn.00, 42Qt.00, 42Qu.00, 42Qv.00, 42h0.00, 66Q..00, 66Q..11, 66Q1.00, 66Q2.00, 66Q3.00, 66Q4.00, 66Q5.00, 66Q6.00, 66Q7.00, 66Q8.00, 66Q9.00, 66QA.00, 66QB.00, 66QC.00, 66QD.00, 66QE.00, 66QF.00, 66QG.00, 88A5.00, B937.14, B937W00, B937W11, D1...00, D10y.00, D10z.00, D11..00, D110.00, D110.11, D110000, D110100, D110200, D110400, D110z00, D111.00, D111000, D111100, D111300, D111400, D111500, D111y00, D111z00, D3...00, D30..00, D30..11, D300.00, D300.11, D300.12, D301.00, D301.11, D301.12, D302.00, D302.11, D302.12, D303.00, D303000, D303100, D303111, D303200, D303300, D303400, D303500, D303600, D303611, D303700, D303800, D303900, D303y00, D303z00, D304.00, D305.00, D305000, D305100, D306.00, D306.11, D306.12, D307.00, D307000, D307100, D307200, D307211, D307y00, D307z00, D308.00, D309.00, D30A.00, D30B.00, D30z.00, D31..00, D310.00, D310000, D310011, D310012, D310100, D310z00, D311.00, D311.11, D311000, D311011, D311z00, D312.00, D312.11, D312.12, D312000, D312100, D312z00, D313.00, D313.11, D313.12, D313.13, D313.14, D313.15, D313000, D313011, D313012, D313100, D313111, D313200, D313211, D313300, D313y00, D313z00, D313z11, D314.00, D314100, D314y00, D314z00, D315.00, D31X.00, D31y.00, D31y000, D31y011, D31yz00, D31z.00, D3y..00, D3y0.00, D3z..00, R027.00, R027.11, R027000, R027z00, TJ42.00, TJ42000, TJ42100, TJ42z00, TJ43.00 | 329 |
| GI symptom or diagnosis  1612.12, 19...00, 19...11, 19...12, 195..00, 195Z.00, 196..00, 196..11, 196..12, 1962, 1963, 1964, 1965, 1965.11, 1968, 1969, 196Z.00, 197..00, 197..11, 197..12, 197..13, 197..14, 1971, 1973, 1974, 1975, 1976, 1977, 1978, 197A.00, 197A.11, 197B.00, 197C.00, 197D.00, 197Z.00, 19A..00, 19A1.00, 19A2.00, 19A3.00, 19A4.00, 19AZ.00, 19Z..00, 19ZZ.00, 25...00, 25...11, 25...12, 251..00, 2511, 2512, 2513, 2514, 2515, 2516, 2516.11, 251Z.00, 258..00, 258..11, 2581, 2582, 2583, 2584, 2584.11, 2585, 2586, 2587, 2587.11, 2587.12, 258Z.00, 259..00, 2591, 2592, 2593, 259Z.00, 25A..00, 25A1.00, 25A2.00, 25A3.00, 25AZ.00, 25B..00, 25B1.00, 25B2.00, 25B3.00, 25B4.00, 25C..00, 25C..11, 25C..12, 25C..14, 25C..15, 25C1.00, 25C2.00, 25C3.00, 25C4.00, 25C5.00, 25C6.00, 25C7.00, 25C8.00, 25C9.00, 25CA.00, 25CZ.00, 25D..00, 25D..11, 25D1.00, 25D2.00, 25D3.00, 25D4.00, 25D6.00, 25D8.00, 25D9.00, 25DA.00, 25DZ.00, 25E..00, 25E1.00, 25E2.00, 25E3.00, 25E5.00, 25E6.00, 25E8.00, 25EA.00, 25EZ.00, 25F..00, 25F1.00, 25F2.00, 25F2.11, 25FZ.00, 25G..00, 25G..11, 25G1.00, 25G2.00, 25G3.00, 25G4.00, 25GZ.00, 25H..00, 25H1.00, 25H2.00, 25H3.00, 25H9.00, 25HA.00, 25HZ.00, 25I..00, 25I1.00, 25I2.00, 25I3.00, 25I5.00, 25I6.00, 25J..00, 25J1.00, 25J2.00, 25J3.00, 25J4.00, 25J5.00, 25J6.00, 25J7.00, 25J8.00, 25J9.00, 25JA.00, 25JZ.00, 25K..00, 25K1.00, 25K2.00, 25K3.00, 25K4.00, 25KZ.00, 25L..00, 25L1.00, 25L2.00, 25LZ.00, 25M..00, 25M1.00, 25M2.00, 25MZ.00, 25N..00, 25N1.00, 25N2.00, 25NZ.00, 25O..00, 25O1.00, 25O2.00, 25O3.00, 25O4.00, 25OZ.00, 25P..00, 25P..11, 25P..12, 25P1.00, 25P2.00, 25P3.00, 25P4.00, 25P5.00, 25P6.00, 25PZ.00, 25Q..00, 25Q..11, 25Q1.00, 25Q5.00, 25Q6.00, 25QZ.00, 25R..00, 25R1.00, 25R2.00, 25R3.00, 25RZ.00, 25S..00, 25S1.00, 25S2.00, 25S3.00, 25S3.11, 25S4.00, 25S5.00, 25SZ.00, 25V..00, 25V0.00, 25Z..00, 3167, 43W9.00, 43WA.00, 4A25.00, 4A26.00, 4JD6.00, 4JM..00, 4JM0.00, 4JM2.00, 4JM3.00, 4JN0.00, 4JO1.00, 68W3.00, 68W4.00, 761H300, 7N30400, 7N30500, 7N30600, 7N33.00, 7N33000, 7N33100, 7N33200, 7N33300, 7N33311, 7N33400, 7N33500, 7N33600, 7N33z00, 7N34.00, 7N34000, 7N34100, 7N34y00, 7N3z.00, J....00, J1...00, J1...11, J1...12, J10y200, J154111, J16y412, J16y600, J344.00, J502100, J521.00, J521.11, J57z.00, J6y..00, J6z..00, Jy...00, R07z.00, R07z.11, R07zz00, R09..00, R090.00, R090000, R090100, R090200, R090300, R090311, R090312, R090400, R090500, R090600, R090700, R090800, R090900, R090A00, R090B00, R090C00, R090D00, R090E00, R090F00, R090H00, R090J00, R090K00, R090N00, R090y00, R090z00, R091.00, R091000, R091z00, R093.00, R093000, R093100, R093111, R093200, R094.00, R095000, R095z00, R096.00, Ryu1.00, Ryu1100, Ryu1200, Ryu1300 | 328 |
| Hospital  13F8.00, 13F8.11, 13F8100, 13F8200, 67IL.00, 67IM.00, 6A1..00, 6A1..11, 8B1..00, 8H...00, 8H1..00, 8H1..11, 8H11.00, 8H12.00, 8H13.00, 8H14.00, 8H2..00, 8H21.00, 8H22.00, 8H24.00, 8H2Z.00, 8H36.00, 8H37.00, 8H39.00, 8H3Z.00, 8H4..00, 8H4..11, 8H4..12, 8H41.00, 8H42.00, 8H47.00, 8H48.00, 8H4D.00, 8H4J.00, 8H4Z.00, 8H4b.00, 8H4l.00, 8H5..00, 8H5..11, 8H51.00, 8H5J.00, 8H5K.00, 8H5Z.00, 8H6..00, 8H61.00, 8H61.11, 8H63.00, 8H64.00, 8H65.00, 8H66.00, 8H68.00, 8H6D.00, 8H6Z.00, 8H7..00, 8H7a.00, 8H7h.00, 8H7o.00, 8HC1.00, 8HE..00, 8HE2.00, 8HEZ.00, 8HF..00, 8HF..11, 8HF..12, 8HM..00, 8HM1.00, 8HM8.00, 8HMG.00, 8HMS.00, 8HMZ.00, 8HN..00, 8HN0.00, 8HN1.00, 8HN2.00, 8HN3.00, 8HN4.00, 8HN5.00, 8HN6.00, 8HN7.00, 8HN8.00, 8HN9.00, 8HNA.00, 8HNB.00, 8HNC.00, 8HND.00, 8HNE.00, 8HNZ.00, 8HO..00, 8HO1.00, 8HO2.00, 8HOZ.00, 8HV0.00, 8HVF.00, 8HVG.00, 8HVM.00, 8HVN.00, 8HVY.00, 8HX..00, 8HX0.00, 8HX1.00, 8HX2.00, 8HY..00, 8HZ..00, 8HZ0.00, 8Ha..00, 8Hb..00, 8Hd..00, 8, Hd0.00, 8Hg5.00, 8Hi..00, 8Hk5.00, 8Hl..00, 8Hl0.00, 8Hm..00, 8Hm1.00, 9N19.00, 9N19.11, 9N1B.00, 9N36.00, 9N36.11, 9N3L.00, 9NC..00, 9NC1.00, 9NC8.11, 9Y...00, 9Y0..00, 9Y1..00, 9Y2..00, ZL16.00, ZL16.11, ZL16100, ZL16111, ZL16200, ZL16211, ZL17.00, ZL18.00, ZL18C00, ZL18D00, ZL18L00, ZL18L11, ZL18R00, ZL18S00, ZL19.00, ZL19100, ZL1A100, ZL1G.00, ZL1GD00, ZL1GD11, ZL1GE00, ZL1GE11, ZL1GF00, ZL1GF11, ZL1GH00, ZL1GJ00, ZL5..00, ZL51.00, ZL51.11, ZL51.12, ZL51.13, ZL52.00, ZL56.00, ZL56.11, ZL56100, ZL56200, ZL56211, ZL57.00, ZL57100, ZL5A.00, ZL5A200, ZL5A211, ZL5AD00, ZL5AE00, ZL5G500, ZL5GA00, ZL5GA11, ZL5GB00, ZL5GB11, ZL5GC00, ZL5GC11, ZL5GE00, ZL9..00, ZL91.00, ZL91.11, ZL91.12, ZL92.00, ZL96.00, ZL96.11, ZL96100, ZL96111, ZL96200, ZL96211, ZL97.00, ZL97100, ZL9A.00, ZL9AE00, ZL9AF00, ZL9AL00, ZL9AL11, ZL9AL12, ZL9G.00, ZL9GC00, ZL9GC11, ZL9GD00, ZL9GD11, ZL9GE00, ZL9GE11, ZL9GG00, ZL9GM00, ZL9GN00, ZL9GP00, ZLD2G00, ZLD2G11, ZLD2H00, ZLD2H11, ZLD2I00, ZLD2I11, ZLD2L00, ZLD2R00, ZLD3.00, ZLD3E00, ZLD3F00, ZLD3L00, ZLD3L11, ZLD4.00, ZLD4700, ZLD4711, ZLD4800, ZLD4811, ZLD4900, ZLD4911, ZLD4A00, ZLD4B00, ZLD4D00, ZLEQ700, ZLEQ711, ZLEQ800, ZLEQ811, ZLEQ900, ZLEQ911, ZLF3.00, ZLG..00, ZLG1.00, ZLG2.00, ZLG3.00, ZLG3100, ZLG3200, ZLG4.00, ZLG4100, ZLG5.00, ZLG5100, ZLG5200, ZLG6.00, ZLG6100, ZLG6300, ZLG6400, ZLG6411, ZLG6500, ZLG6511, ZLG8.00 | 294 |
| Collapse  1479, 147A.00, 147B.00, 147C.00, 147D.00, 16D..00, 16D1.00, 16D5.00, 1B6..00, 1B6..11, 1B6..12, 1B6..13, 1B62.00, 1B65.00, 1B65.11, 1B66.00, 1B66.11, 1B68.00, 2225, 2235, 2236, 2236.11, 2236.12, 2236.13, 2236.14, 2237, 2238, 2239, 223Z.00, 224..00, 2241, 2242, 2243, 2244, 224Z.00, C365.00, C365000, C365100, C365200, C365z00, G575.00, G575.11, G575.12, G575000, G575100, G575200, G575300, G575z00, G87..00, G870.11, R000.00, R000.11, R000.12, R000000, R000200, R000300, R000311, R000400, R000500, R000z00, R002.00, R002.11, R002000, R002100, R002200, R002300, R002400, R002500, R002600, R002700, R002z00, R003.00, R003000, R004000, R004100, R004200, R055.00, R055000, R055011, R055100, R055111, R200.12, R2y0.00, R2y0100, R2y1.00, R2y1000, R2y1100, R2y1z00, SP20.11, U10..00, U100.00, U100000, U100200, U100300, U100400, U100500, U100z00, U101.00, U101000, U101100, U101200, U101300, U101400, U101500, U101600, U101700, U101y00, U101z00, U102.00, U102000, U102300, U102400, U102700, U102y00, U102z00, U103.00, U103000, U103300, U103y00, U103z00, U104.00, U104000, U104100, U104600, U105.00, U105000, U105100, U105500, U105700, U106.00, U106000, U106100, U106200, U107.00, U107000, U107200, U107600, U107y00, U107z00, U108.00, U108000, U108100, U108600, U108z00, U109.00, U109000, U109200, U109z00, U10A.00, U10A000, U10A100, U10A400, U10A500, U10A511, U10Ay00, U10Az00, U10B.00, U10B000, U10B600, U10By00, U10Bz00, U10C.00, U10C600, U10Cz00, U10D.00, U10D000, U10D100, U10D300, U10D600, U10Dz00, U10E.00, U10E000, U10Ez00, U10F000, U10F100, U10F300, U10G.00, U10G300, U10G600, U10H.00, U10H000, U10H200, U10H300, U10H400, U10H500, U10H600, U10Hy00, U10Hz00, U10J.00, U10J000, U10J100, U10J200, U10J300, U10J400, U10J600, U10Jy00, U10Jz00, U10z.00, U10z000, U10z100, U10z300, U10z400, U10z600, U10zy00, U10zz00 | 280 |
| Alcohol  136..00, 1361.11, 1363, 1364, 1365, 1366, 1368, 1369, 136C.00, 136D.00, 136E.00, 136F.00, 136G.00, 136H.00, 136I.00, 136J.00, 136K.00, 136L.00, 136O.00, 136P.00, 136Q.00, 136R.00, 136S.00, 136T.00, 136V.00, 136W.00, 136X.00, 136Z.00, 13Y8.00, 1462, 1B1c.00, 2577, 2577.11, 66e..00, 66e0.00, 6792, 67H0.00, 8H35.00, 8H7p.00, 8HkG.00, E01..00, E010.00, E010.11, E010.12, E011.00, E011000, E011100, E011200, E011z00, E012.00, E012.11, E012000, E013.00, E014.00, E014.11, E015.00, E01y.00, E01y000, E01yz00, E01z.00, E23..00, E23..11, E23..12, E230.00, E230.11, E230000, E230100, E230200, E230300, E230z00, E231.00, E231.11, E231000, E231100, E231200, E231300, E231z00, E23z.00, E250.00, E250.11, E250.12, E250.13, E250.14, E250000, E250100, E250200, E250300, E250z00, Eu10.00, Eu10000, Eu10011, Eu10100, Eu10200, Eu10211, Eu10212, Eu10213, Eu10300, Eu10400, Eu10411, Eu10500, Eu10511, Eu10512, Eu10513, Eu10514, Eu10600, Eu10611, Eu10700, Eu10711, Eu10712, Eu10800, Eu10y00, Eu10z00, F375.00, J610.00, J611.00, J613.00, J613000, J617.00, J617000, J671000, R103.00, U80..00, U800.00, U801.00, U802.00, U803.00, U804.00, U805.00, U806.00, U807.00, U808.00, U81..00, U811.00, U812.00, U813.00, U814.00 | 251 |
| Upper GI diagnosis  43k7.00, 4JM1.00, 4JN..00, A074500, AB20100, AB20z00, J10..00, J100.00, J100.11, J100.12, J100000, J101000, J103000, J103100, J103200, J103211, J103300, J103311, J105.00, J105.11, J105.13, J105.14, J105.15, J105000, J106.00, J106000, J106100, J106200, J106300, J106400, J106500, J106z00, J10y.00, J10y100, J10y413, J160.00, J161.00, J170.00, J170.11, J170000, J170100, J170200, J170z00, J171.00, J172.00, J174000, J175.00, J176.00, J17y.00, J17y000, J17y100, J17y300, J17y500, J17y600, J17yz00, J17z.00, J1y..00, J1z..00, J34..00, J34..11, J34..12, J340.00, J341.00, J342.00, J343.00, J347.00, J348.00, J34y.00, J34y.11, J34z.00, J34z000, Jyu1000, Jyu1400, PA30.00, PA31.00, PA31.11, PA32.00, PA32000, PA32100, PA32111, PA32z00, PA33.00, PA34.00, PA35.00, PA36.00, PA37.00, PA3y.00, PA3z.00, PA4..00, PA40.00, PA42.00, PA43.00, PA44.00, PA45.00, PA4z.00, PA5..00, PA50.00, PA51.00, PA51.11, PA52.00, PA52.11, PA5y.00, PA5z.00, PA6..00, PA7..00, PA70.00, PA70.11, PA71.00, PA73.00, PA74.00, PA75.00, PA76.00, PA77.00, PA78.00, PA7z.00, PAy..00, PAz..00, PAz0.00, PAz1.00, PAz2.00, PAzz.00, PAzz.11, PB13000 | 152 |
| Confusion  1B67.00, 1B67.11, 1B69.00, 1B6A.00, 2232, 2232.11, 2233, 2234, 225..00, 225..11, 2251, 2841, 2841.11, E030.00, E030.11, E030.12, E030000, E030100, E030200, E030300, E030400, E030z00, E031.00, E031.11, E031000, E031z00, Eu04.12, R009.00, R009.11, R009000 | 92 |
| Upper GI procedure  7022000, 7022012, 7022100, 7022111, 7022112, 7022200, 7022300, 7022400, 7022y00, 7022z00, 7022z11, 76...00, 76...11, 760..00, 760..11, 7600, 7600.11, 7600000, 7600011, 7600012, 7600013, 7600100, 7600111, 7600300, 7600y00, 7600z00, 7601, 7601.11, 7601000, 7601111, 7601200, 7601213, 7601400, 7601y00, 7601z00, 7602, 7602.11, 7602000, 7602300, 7602y00, 7602z00, 7602z11, 7603, 7603000, 7603100, 7604, 7604000, 7604100, 7604300, 7604500, 7604z00, 7605, 7605000, 7605100, 7605200, 7605y00, 7606, 7606200, 7606300, 7606y00, 7606z00, 7607, 7607.11, 7607000, 7607200, 7607211, 7607300, 7607y00, 7607z00, 7607z11, 7608, 7608000, 7608011, 7608100, 7608200, 7608300, 7608311, 7608y00, 7608z00, 7609, 7609000, 7609100, 7609200, 7609400, 7609y11, 7609z00, 760A.00, 760A.11, 760A000, 760A011, 760A100, 760A200, 760B.00, 760B000, 760B100, 760By00, 760Bz00, 760Hz00, 760J.00, 760J300, 760J312, 760J500, 760Jy00, 760Jz00, 760K.00, 760y.00, 760z.00, 761..00, 761..11, 7610, 7610.11, 7610.12, 7610000, 7610100, 7610300, 7610400, 7610y00, 7610z00, 7611, 7611.11, 7611000, 7611011, 7611012, 7611100, 7611200, 7611211, 7611212, 7611213, 7611214, 7611215, 7611216, 7611300, 7611400, 7611500, 7611600, 7611700, 7611800, 7611900, 7611A00, 7611y00, 7611z00, 7612, 7612000, 7612100, 7612111, 7612200, 7612300, 7612400, 7612500, 7612y00, 7614, 7614000, 7614100, 7614111, 7614200, 7614y00, 7614z00, 7615, 7615.11, 7615000, 7615100, 7615200, 7615y00, 7615z00, 7616, 7616000, 7616011, 7616012, 7616013, 7616014, 7616015, 7616100, 7616200, 7616300, 7616600, 7616y00, 7616z00, 7617, 7617.11, 7617.12, 7617000, 7617111, 7617112, 7617200, 7617300, 7617500, 7617y00, 7617z00, 7618, 7618000, 7618100, 7618200, 7618y00, 7618z00, 7619, 7619.11, 7619000, 7619100, 7619y00, 7619z00, 761A.00, 761A000, 761A100, 761A200, 761A300, 761A400, 761Ay00, 761Az00, 761B.00, 761B.11, 761B000, 761B011, 761B100, 761B200, 761B211, 761B212, 761B213, 761B300, 761B500, 761B600, 761By00, 761Bz00, 761C.00, 761C000, 761C100, 761Cy00, 761Cz00, 761Gy00, 761Gz00, 761Hy00, 761Hz00, 762..00, 7620, 7620.11, 7620000, 7620100, 7620200, 7620y00, 7620z00, 7620z11, 7621000, 7621100, 7621z00, 7622, 7622100, 7622200, 7622y00, 7622z00, 7623, 7623000, 7623100, 7623200, 7623300, 7623400, 7623411, 7623500, 7623700, 7623y00, 7623z00, 7624, 7626, 7626100, 7626y00, 7626z00, 7627y00, 7627z00, 762y.00, 762z.00, 8HS..00, 8HS..11, J522.00, J522000, J522100, J522200, J522211, J522212, J522z00, J523.00, J524100 | 39 |
| Blood transfusion  14S1.00, 4311, 434..00, 4341, 4342, 4343, 434Z.00, 435..00, 435..11, 7L13.00, 7L13000, 7L13100, 7L13200, 7L13300, 7L13500, 7L13y00, 7L13z00, 7L14.00, 7L14000, 7L14100, 7L14200, 7L14300, 7L14311, 7L14y00, 7L14z00, 7L15.00, 7L15000, 7L15100, 7L15200, 7L15300, 7L15400, 7L15800, 7L15y00, 7L15z00, 7L16.00, 88...11 | 36 |
| GI procedure  14U2.00, 14U5.00, 1984.11, 585F.00, 7603300, 7603311, 7606000, 7606011, 7606100, 760D312, 760J000, 760J100, 760J200, 760K.11, 760K.12, 760K000, 760K011, 760K012, 760K100, 760K200, 760K300, 760K400, 760K500, 760Ky00, 760Kz00, 760L.00, 760L.11, 760L000, 760L011, 760L012, 760L100, 760L111, 760L200, 760L211, 760L300, 760L311, 760L312, 760L500, 760L600, 760L611, 760L700, 760L800, 760Ly00, 760Lz00, 760M.00, 760M000, 760M200, 760Mz00, 7613, 7613000, 7613100, 7613111, 7613200, 7613300, 7613400, 7613500, 7613600, 7613y00, 7613z00, 7617100, 7617400, 7617600, 761A500, 761E400, 761E900, 761EA00, 761G000, 761G100, 761G212, 761G300, 761H.00, 761H000, 761H100, 761H200, 7623711, 7626000, 782B.00, 782B.11, 782B000, 782B011, 782B100, 782B111, 782By00, 782Bz00, 782C.00, 782C000, 782Cy00, 782Cz00, 782D.00, 782D000, 782D100, 782D200, 782D300, 782D400, 782D500, 782D600, 782Dy00, 782Dz00, 782E.00, 782E000, 782E100, 782E200, 782Ey00, 782Ez00, 782F.00, 782F000, 782F100, 782F200, 782F300, 782F400, 782Fy00, 782Fz00, 782G.00, 782G000, 782G100, 782G200, 782Gy00, 782Gz00, 782Gz11, 782Gz12, 782H.00, 782H000, 782Hy00, 782Hz00, 782J.00, 782J000, 782J100, 782Jy00, 782Jz00, 782K.11, 782Kz00, 782L.00, 782L300, 782L400, 782Ly00, 782Lz00, 782M.12, 782M000, 782M100, 782M200, 782M400, 782M500, 782Mz00, 782N.00, 782N000, 782Nz00, J16..00, J173.00, J173100, J173200, J173300, J173z00, J174.00, J174100, J174200, J174300, J174400, J174z00, J177.00, J177.11, J178.00, J178.11, J17y200, J17y400, J17y700, J345.00, J346.00, J500000, J500100 | 34 |
| Nutrition  161..00, 1612, 1612.11, 1623, 1625, 1625.11, C2...00, C20..00, C201.00, C20z.00, C20zX00, C21..00, C22..00, C23..00, C230.00, C231.00, C232.00, C233.00, C234.00, C23y.00, C23z.00, C23z.11, C23z.12 | 25 |
| General care  1GZ..00, 1H...00, 1H0..00, 1H0..11, 1H1..00, 1H2..00, 1H3..00, 1I...00, 1J0..00, 1R...00, 1R0..00, 1R1..00, 431..00, 431..11, 4312, 4313, 4314, 4315, 431Z.00, 432..00, 4321, 4322, 4323, 4324, 4325, 432Z.00, 433..00, 4331, 4332, 4333, 4334, 4334000, 4335, 4336, 4337, 4338, 433Z.00, 4344, 436..00, 4361, 4362, 436Z.00, 437..00, 437..11, 4371, 4372, 4373, 4374, 4375, 4376, 437Z.00, 43S..00, 43S0.00, 43c0.00, 43x0.00, 43x1.00, 43x2.00, 43x3.00, 43x4.00, 43x5.00, 43x6.00, 62L..00, 62L2.00, 62LZ.00, 6A...00, 8CB..00, 8H3U.00, ZLG6200 | 15 |
| General procedure  89...00, 89...11, 89...12, 89...13, 89...14, 891..00, 892..00, 8920, 8921, 8922, 8923, 893..00, 8934, 8935, 89Z..00, 8A...00, 8A1..00 | 15 |
